# Dental Enactments

**Published:** 1896-02

**Authors:** 


ARTICLE VIII.

### DENTAL ENACTMENTS.

Every one who has had anything to do with dental legislation must be impressed with the great unanimity among practitioners regarding the duty of some other man's making the necessary complaint. It reminds one of

Swift's definition of the charitable spirit: "A no sooner sees B in distress, than he petitions C. to relieve him." Those who have never paid a dollar or given a moment's time to the procurement or support of dental legislation, are most anxious for the repression of irregular practitioners, and loudest in their complaints of unlicensed competitors. They write importunate letters to those who have for many years devoted themselves to dental reform, and always with the charge that their names are not to be used. They will not sign a formal complaint, or assist in obtaining testimony against an illegal practitioner, demanding that those who have armed them with weapons for their own protection shall also fight their battles. It is very seldom the case that the dentists who have labored to establish a reputable profession, and to prevent the intrusion of unqualified men, can in any why personally profit by their labors, except indirectly. Their status is usually secure, and they have nothing personally to fear from illegal or unqualified competitors. Their work is for their profession, and their labors are unselfish. But so accustomed are others to rely upon their generosity, that unless they at once drop their own business to harry some interloper, they are roundly abused for declining to be at the beck and call of those who would use them to compass their own ends.

There can be no advance made without the co-operation of all. If it is desirable to draw the lines between legal and illegal practitioners, and to repress those who are unqualified for practice, there must be the active support and sympathy of every man who regards his profession. There must be a general determination on the part of all qualified dentists to enforce the law, and complaints should be made against every one who practises illegally. The inspiration for this should come, not from any personal jealousy or sentiment of rivalry, but from a genuine love for and desire to advance the best interests of the profession. If those who are to be benefitted by legislation give it but a half-support, public sentiment is influenced and the law cannot be enforced. It is the bounden duty of every professional man to assist in upholding the law, not only in his own neighborhood, but everywhere. He should strengthen the hands of those who are charged with the enforcement of the enactments, not alone by monetary contributions, for there are few who will give a dollar, but by words and acts. Unless there is unanimity among dentists, the public sentiment upon which all must depend for the support of the law will certainly condemn it.—*The Dental Practitioner and Advertiser.*

